# Bacteriophage-based particles carrying the TNF-related apoptosis-inducing ligand (TRAIL) gene for targeted delivery in hepatocellular carcinoma†

**DOI:** 10.1039/d3nr05660k

**Published:** 2024-02-23

**Authors:** Pattaralawan Sittiju, Benjawan Wudtiwai, Aitthiphon Chongchai, Amin Hajitou, Prachya Kongtawelert, Peraphan Pothacharoen, Keittisak Suwan

**Affiliations:** a Thailand Excellence Center for Tissue Engineering and Stem Cells, Department of Biochemistry, Faculty of Medicine, Chiang Mai University Chiang Mai Thailand peraphan.pothacharoen@gmail.com; b Cancer Phage Therapy Group, Department of Brain Sciences, Faculty of Medicine, Imperial College London London UK ksuwan@imperial.ac.uk

## Abstract

The TRAIL (Tumour Necrosis Factor-Related Apoptosis-Inducing Ligand) is a promising candidate for cancer treatment due to its unique ability to selectively induce programmed cell death, or apoptosis, in cancer cells while sparing healthy ones. This selectivity arises from the preferential binding of the TRAIL to death receptors on cancer cells, triggering a cascade of events that lead to their demise. However, significant limitations in using the TRAIL for cancer treatment are the administration of the TRAIL protein that can potentially lead to tissue toxicity (off-target) and the short half-life of the TRAIL in the body which may necessitate frequent and sustained administration; these can pose logistical challenges for long-term treatment regimens. We have devised a novel approach for surmounting these limitations by introducing the TRAIL gene directly into cancer cells, enabling them to produce the TRAIL locally and subsequently trigger apoptosis. A novel gene delivery system such as a bacteriophage-based particle TPA (transmorphic phage/AAV) was utilized to address these limitations. TPA is a hybrid M13 filamentous bacteriophage particle encapsulating a therapeutic gene cassette with inverted terminal repeats (*ITRs*) from adeno-associated viruses (AAVs). The particle also showed a tumour targeting ligand, CDCRGDCFC (RGD4C), on its capsid (RGD4C.TPA) to target the particle to cancer cells. RGD4C selectively binds to α_v_β_3_ and α_v_β_5_ integrins overexpressed on the surface of most of the cancer cells but is barely present on normal cells. Hepatocellular carcinoma (HCC) was chosen as a model because it has one of the lowest survival rates among cancers. We demonstrated that human HCC cell lines (Huh-7 and HepG2) express α_v_β_5_ integrin receptors on their surface. These HCC cells also express death receptors and TRAIL-binding receptors. We showed that the targeted TPA particle carrying the transmembrane *TRAIL* gene (RGD4C.TPA-*tmTRAIL*) selectively and efficiently delivered the *tmTRAIL* gene to HCC cells resulting in the production of tmTRAIL from transduced cells and subsequently induced apoptotic death of HCC cells. This tumour-targeted particle can be an excellent candidate for the targeted gene therapy of HCC.

## Abstract 2

The TRAIL (Tumour Necrosis Factor-Related Apoptosis-Inducing Ligand) is a promising candidate for cancer treatment due to its unique ability to selectively induce programmed cell death, or apoptosis, in cancer cells while sparing healthy ones.

## Graphical abstract

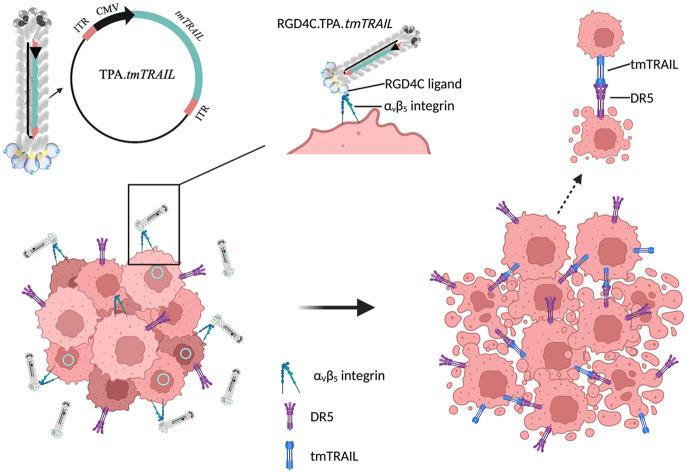

## Introduction

The tumour necrosis factor-related apoptosis-inducing ligand (TRAIL) is a prospective molecule that uses human natural mechanisms to induce apoptosis, or programmed cell death, in cancer cells. It has the advantage of inducing cell death in a highly selective manner, allowing it to target cancer cells while sparing normal cells.^1,2^ This selectivity reduces the adverse reactions that are frequently associated with standard cancer treatments.^1^ The TRAIL has also shown promise in overcoming resistance to chemotherapy and radiation therapy. The major limitations of using the TRAIL in cancer treatment are its relatively short half-life and rapid clearance from the blood circulation, limiting its effectiveness in reaching tumors at therapeutic levels.^3–5^ This short half-life requires repeated and prolonged administration of the TRAIL, which can be challenging in terms of patient compliance and potential toxicity.^3^ Overcoming these challenges requires ongoing research to develop strategies that enhance the therapeutic potential of the TRAIL.

Gene therapy is an alternative strategy for cancer treatment. By introducing or modifying genes within cancer cells, it aims to correct or replace the genetic information of cancer cells, effectively thwarting the uncontrolled cell growth characteristic of cancer or inducing tumor cell death.^6^ This approach represents a cutting-edge advancement with the potential to revolutionize how we combat cancer at its genetic roots. Several studies have evaluated the safety and efficacy of TRAIL gene therapy in patients with advanced stages of cancer.^7–9^ However, cancer gene therapy faces challenges primarily due to a lack of cell specificity. Many traditional gene delivery methods struggle to exclusively target cancer cells, leading to unintentional effects on healthy tissue. This lack of precision can result in off-target effects and unintended consequences, limiting the effectiveness and safety of the therapy.^6^ Achieving high levels of cell specificity remains a crucial area of research in order to unlock the full potential of gene therapy in cancer treatment.

We previously reported a Transmorphic Phage/AAV (TPA) particle to target cancers using the harmless and non-pathogenic filamentous M13 bacteriophage (phage) as a vector to convey therapeutic genes.^10^ This particle was engineered to contain mammalian gene cassettes flanked by the human adeno-associated virus (AAV2) inverted terminal repeats (*ITRs*), which are crucial for this delivery platform.^11,12^ We employed a phagemid-based system to remove all phage genes from TPA, while retaining the origin of the replication of the phage, *f1 ori*, to allow the replication of the single-stranded TPA DNA in bacteria and its packaging by the phage coat proteins provided by a helper phage infection. This resulted in a much shorter TPA particle with improved trafficking in mammalian cells, increased vector uptake and subsequently enhanced gene delivery.^10^ It is important to highlight that the helper phage coat proteins exhibited the double cyclic CDCRGDCFC (RGD4C) ligand on the pIII coat proteins for tumour targeting, resulting in a compact vector containing only the therapeutic genetic payload for delivery to the target cells. Following the entry of the phage particles into the cells, the mammalian *ITR*-flanked transgene expression cassette is released to allow the transgene to be expressed in tumours through the cytomegalovirus (*CMV*) promoter.^2,11–13^ This particle subsequently provides significant enhancement of gene delivery compared to a phage vector containing unnecessary phage genes. The double cyclic RGD4C peptides on the pIII capsid of TPA particles can target the particle specifically to α_v_β_3_ and α_v_β_5_ integrin-expressing cancer cells. Both integrins are specific binding partners to the RGD4C ligand, facilitating the subsequent delivery of the transgene to these cells. In contrast, the TPA vector without RGD4C on its capsid failed to target and deliver the transgene to cancer cells. This was confirmed in both *in vitro* and *in vivo* experiments. The intravenous delivery of our phage-based particle exhibiting the RGD4C peptide to various animal models of tumours resulted in particle accumulation at the tumour site, but not in other organs. Transgene expression was observed only in the tumour tissue.^10,14–16^

We chose to test the tumour-targeted phage-based particle carrying the TRAIL gene in hepatocellular carcinoma (HCC) models. HCC is a major public health concern around the world with differing rates of occurrence in different regions. It is the fourth-highest cause of cancer-related mortality worldwide according to the World Health Organization (WHO).^17,18^ It is estimated that over 800 000 new cases of liver cancer are diagnosed each year, with HCC accounting for 75–85% of all cases.^17^ Depending on the stage of malignancy, treatment options for HCC may include surgery, liver transplantation, localised therapies (such as ablation or embolisation), and systemic therapies (such as targeted therapy or immunotherapy).^13,19^ However, the prognosis of HCC remains poor, with a low overall survival rate, particularly in advanced-stage tumours.^13,17–19^ From our meta-analysis, high expression of α_v_ and β_5_ integrins was observed in HCC, both in primary tumour cells. HCC cell lines (HepG2 and Huh-7) also express α_v_β_5_ on their cell surface. The involvement of the α_v_β_5_ integrin in all these events, along with its high expression in HCC tissues, is also well reported.^20^ This makes HCC a suitable model for targeted gene therapy mediated by the TPA vector. In this study, we generated a hepatocellular carcinoma-targeted particle using our tumour-targeted TPA platform. The particle carries a therapeutic TRAIL gene expression cassette packaged inside the phage capsid. We demonstrated that tumour-targeted TPA effectively targeted and enabled TRAIL transgene expression in HCC cells, both in 2D and tumoursphere models. Importantly, no transfer of the TRAIL transgene was detected in a normal liver cell model. Subsequently, the production of the TRAIL transgene by HCC itself led to the induction of apoptotic death of HCC cells.

## Experimental

### Construction and production of superior bacteriophage-based particles

**Fig. 1:**
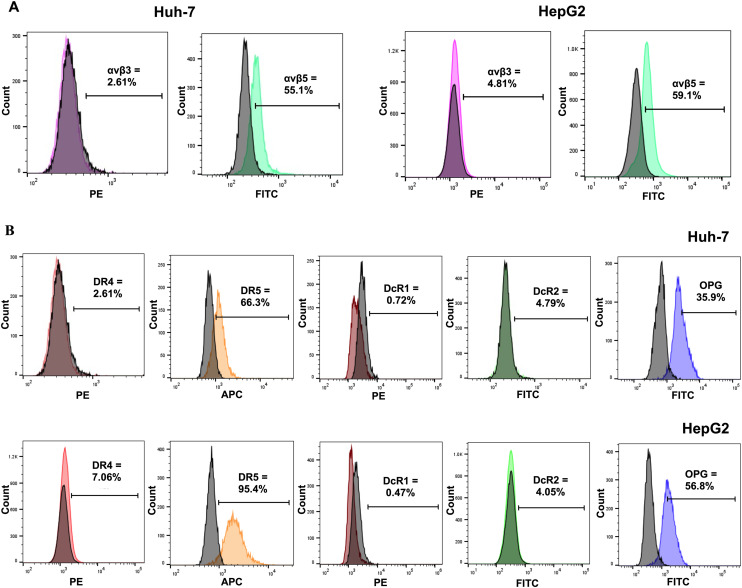
Expression of integrins (α_v_β_3_ and α_v_β_5_) and TRAIL death receptors on human HCC cells. Histograms of flow cytometric analysis of integrins (A) and TRAIL receptors (B) from Huh-7 and HepG2 cells. The black histograms represent unstained or isotype control staining cells.

To generate the tumour-targeted Transmorphic Phage/AAV (TPA) particle carrying the TRAIL transgene, we used a phagemid-based system. We engineered the filamentous M13 phage genome to exhibit the RGD4C ligand, a tumour-targeting peptide, on pIII minor coat proteins.^10^ In brief, M13KO7 was modified to insert the double cyclic RGD4C DNA sequence on the pIII minor coat protein gene using DNA primers and then amplified using Q5 High-Fidelity DNA Polymerase (NEB, UK). Subsequently, the amplicon was self-ligated using a quick ligase (NEB, UK) and transformed into chemically competent Mix&Go Competent TG1 *E. coli* (Zymo Research, US). The transformed cells were selected by plating on tryptone yeast extract (TYE) agar supplemented with 50 μg mL^−1^ kanamycin and then incubated for 18 hours at 37 °C. The colonies were picked and grown in a 2×YT medium, supplemented with 50 μg mL^−1^ kanamycin by shaking at 200 rpm overnight for another 18 hours. Next, bacterial outgrowth was extracted using the plasmid miniprep kit (Qiagen, UK) to obtain DNA. The extracted DNA was run on gel electrophoresis to demonstrate the actual plasmid size, then subjected to the sequencing (Eurofins, Germany) of the pIII gene to certify positive clones. This particle acts as a helper phage. The TPA (phagemid) was modified to incorporate a transgenic expression cassette. The cassette contains a *CMV* promoter, a transgene, and AAV2 *ITR cis* elements (Fig. 4A). The *CMV* promoter-controlled DNA sequence encoding the transmembrane form of TRAIL was introduced into the transgenic expression cassette (Fig. 4A). To clone the TRAIL DNA fragment into the cassette, the TRAIL DNA sequence (a full form of TRAIL gene, 287 amino acids) was inserted into the TPA vector with *EcoRI* and *SalI* restriction sites. For TPA production, the plasmid of the TPA vector was transformed into the competent *E. Coli* TG1 cells (Zymo Research, USA) according to the manufacturer's protocol. The transformed bacteria were grown to the log phase (OD_600_ = 0.4–0.6) in 50 mL of 2×YT medium with 100 μg mL^−1^ ampicillin at 32 °C, 180 rpm, and a 1 × 10^12^ transduction unit (TU) helper phage RGD4C.M13KO7 or M13KO7 phage was added, mixed by swirling, and incubated (37 °C) without shaking for 30 minutes. The culture was then added to 450 mL fresh 2×YT medium supplemented with 100 μg mL^−1^ ampicillin and 50 μg mL^−1^ kanamycin, and incubated overnight for 18 hours, shaking at 180 rpm, 37 °C. Next, the overnight bacterial culture was centrifuged twice at 6000*g* to remove bacteria from the culture supernatant. The vectors in the supernatant were precipitated twice with a final concentration of 30% (w/v) polyethylene glycol (PEG)/NaCl. The precipitate phage particles were isolated by centrifugation at 10 000*g* for 30 minutes. Finally, a pellet of the TPA vector was dissolved in 2 mL PBS and subsequently passed through a sterile-filtered 0.45 μm low protein binding PVDF filter. The TPA production workflow is shown in the ESI, Fig. 1.† The titer of TPA was determined as functional bacterial transducing units (TUs) by colony counting of host bacteria as described by Hajitou A., *et al.*^21^

### Cell culture

Human HCC cell lines: Huh-7, HepG2, and liver cell line, LX-2, were obtained from the American Type Culture Collection (ATCC, US). The cells were grown in 10% fetal bovine serum Dulbecco's Modified Eagle's medium (DMEM) and maintained under a humidified atmosphere of 37 °C with 5% CO_2_. Cell lines were regularly tested for mycoplasma with a mycoplasma detection kit (Lonza, UK).

### Tumoursphere formation assay

100 mg mL^−1^ poly-HEMA (2-hydroxyethyl methacrylate) solution was prepared in 95% ethanol. 50 μL of solution was added into each well of a 96-well U-bottomed cell culture plate. After the plates were completely dried, the HCC cell line was seeded into each well at a concentration of 5 × 10^4^ cells per mL. The plates were centrifuged at 216*g* at room temperature (15–20 °C) for 10 minutes and incubated for 18 hours at 37 °C, 5% CO_2_.

### Flow cytometry

All HCC cell lines were harvested using cell dissociation buffer (Life Technologies, UK) and washed twice with phosphate-buffered saline (PBS). Cell numbers were adjusted to 1 × 10^6^ cells per mL in PBS. The harvested cells were stained with either heterodimer phycoerythrin (PE)-conjugated anti-human α_v_β_3_ (diluted 1 : 100) or fluorescein isothiocyanate (FITC)-conjugated anti-human α_v_β_5_ (diluted 1 : 25) antibodies (Merck, Germany) diluted with 5% bovine serum albumin (BSA) in PBS (Thermo Fisher Scientific, UK) for 1 hour at 4 °C in the dark, then washed three times with washing buffer (0.5% BSA in PBS). For death receptor (DR) expression, the cells were stained with primary antibodies against PE-conjugated anti-human DR4 (BioLegend, US), allophycocyanin (APC)-conjugated anti-human DR5 (BioLegend, US), PE-conjugated anti-human decoy receptor-1 (DcR1) (Miltenyi biotec, Germany), or anti-human DcR2 (Invitrogen, UK) with dilutions of 1 : 25, 1 : 100, 1 : 50, and 1 : 50, respectively, in 5% BSA in PBS for 1 hour at 4 °C. The samples were kept in the dark and washed three times with washing buffer. For osteoprotegerin (OPG) staining, the cells were permeabilized with 0.1% saponin in 1% BSA-PBS for 1 hour at room temperature before staining with primary antibodies against human OPG diluted 1 : 100 (Novus Biologicals, US). The cells were subsequently incubated with the secondary antibody, goat anti-rabbit IgG AlexaFluor-488 (diluted 1 : 1000). Prior to analysis, the cells were washed twice with washing buffer and resuspended in PBS. Isotype-antibody or secondary antibody-stained cells were used as controls. Subsequently, integrin and death receptor expression was measured by flow cytometry and analyzed using the FlowJo software (BD Biosciences, US).

### *In vitro* transfection using the TPA-*tmTRAIL* plasmid

The HCC cells were seeded in a 6-well culture plate format and grown for 24 hours to reach 70% confluence. Culture medium was change to reduced-serum medium for 2 hours (Opti-MEM, Thermofisher, UK) prior transfection. The transfection mixture was prepared using 2 μg of TPA-*tmTRAIL* plasmid to 6 μL of FuGENE® HD (Promega, UK) in reduced-serum medium. The mixture was incubated for 20–25 minutes at room temperature. Next, the mixture was added dropwise to the culture plate containing cells in reduced-serum medium. The cells were again incubated for 18 or 48 hours. Finally, the cell lysate was collected for further analysis.

### *In vitro* transduction using the TPA particle

The HCC were seeded in appropriate size culture plate to achieve 70–80% confluence 48 hours after seeding. The average number of cells per well or plate culture was calculated the day of transduction and was used to calculate how much TPA particles to add to the cells. Generally, one million TU of TPA were added to one cell. The appropriate amount of the particle stock solution is then diluted in serum-free medium, and thoroughly mixed to prepare the transduction mixture. The smallest amount required to completely cover the cell monolayer is the suggested volume of transduction mixture used per well. The transduced cells were cultured until they were analysed.

### Cell death assays

#### MTT assay

The 3-[4,5-dimethylthiazol-2-yl]-2,5 diphenyl tetrazolium bromide (MTT) assay was used to measure cell viability. HCC cells were seeded into 96-well culture plates in DMEM medium. At the end of each experiment, the treated cells were cultured for 4 hours at 37 °C in a CO_2_ incubator with the MTT dye (Sigma-Aldrich, US). After removing the supernatant, the formazan crystals were dissolved in dimethyl sulfoxide (DMSO). A spectrophotometric plate reader at 540 nm and a reference wavelength of 630 nm were used to quantify the absorbance of dissolved formazan. Cell viability was calculated as
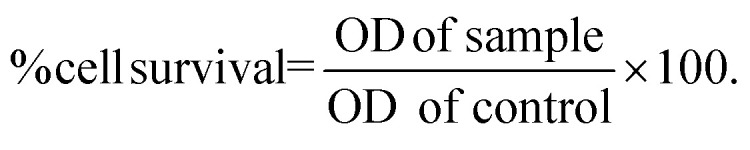


#### Alamar Blue assay

Alamar Blue assay was performed in a 96-well plate. Following treatment, 10% v/v Alamar Blue (Sigma-Aldrich, US) was added to each well after the culture media were aspirated. The cells were incubated for 4 hours at 37 °C. Finally, the fluorescence intensity was evaluated using a Synergy™ H4 Hybrid Multi-Mode Microplate Reader. The detection was carried out at 530 nm excitation and 590 nm emission wavelengths. The association of fluorescence intensity with the rate of decrease in cellular metabolic activity was measured as cell viability. To produce the 100% reduced form of Alamar Blue, simply autoclave a media containing 10% v/v Alamar Blue for 15 minutes. The percentage of cell viability was estimated using the formula:
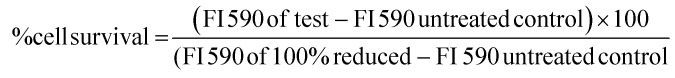
FI 590 = Fluorescence intensity at 590 nm emission (530 nm excitation)

### Luciferase reporter gene assay

To optimise the amount of the TPA particle for transduction, 60–70% confluence cells were transduced with different amounts of the TPA particle (1 × 10^5^ to 2 × 10^6^ TU per cell) carrying secreted luciferase (*lucia*) transgenes, including (1) M13.TPA-*lucia* for the non-targeted particle and (2) RGD4C.TPA-*lucia* for the targeted particle. The cells were cultured at 37 °C in a 5% CO_2_ humidified environment for 24 hours. Next, the medium was changed to DMEM supplemented with 10% (v/v) FBS and incubated at 37 °C to facilitate the transgene expression. The culture medium was collected from day 3 to day 7 after transduction for the determination of *lucia* expression using the QUANTI-Luc™ detection kit (InvivoGen, France). The procedure was carried out according to the manufacturer's procedure. GloMax® Navigator Microplate Luminometer (Promega, UK) was used to measure luminescence.

### RNA purification and RT-qPCR

Total RNA was extracted with an Illustra RNAspin Mini Kit (GE Healthcare Europe GmbH, Freiburg, Germany) and 1 μg of cDNA were generated using iScript™ (Bio-Rad, Hercules, CA, US). PCR was performed with an Applied Biosystems 7500/7500 Fast Real-Time PCR system using SensiFAST™ SYBR® Lo-ROX (Bio-Rad, Hercules, CA, US) for 45 cycles. Each cycle was performed as follows: 5 seconds at 95 °C, 10 seconds at 60 °C and then 30 seconds at 72 °C. The RNA levels were normalised with the β-actin gene (*ACTB*) as a housekeeping gene using the 2 ^(−ΔΔC (T))^ method. Primer sequences are listed in the ESI, Table 1.†

### ELISA for TRAIL quantification

After treatment, the cell lysates of the treated cells were extracted using radioimmunoprecipitation assay (RIPA) buffer. The amount of the transmembrane TRAIL in the cell lysate was measured using a Human TRAIL/TNFSF10 DuoSet ELISA kit (R&D systems, UK). Briefly, the captured antibody was coated on ELISA plates and incubated overnight at room temperature. Next, the plates were then blocked with a reagent diluent at room temperature for 1 hour. Standards and samples were added into the plate and incubated at room temperature for 2 hours. The detection antibody in reagent diluent with 2% heat inactivated normal goat serum (goat) solution was added and incubated at room temperature for 2 hours. Next, diluted Avidin-HRP was added and incubated for 20 minutes. The HRP substrate solution was added to the plates to determine the TRAIL quantity. Finally, the reaction was stopped by adding 2 N H_2_SO_4_ solution and absorbance was measured at 450 nm. At the end of every step, the plate was washed three times with washing buffer (0.05% Tween20 in PBS).

### Western blotting

The treated cells were collected and lysed in RIPA buffer containing protease and phosphatase inhibitors. The Bradford assay was used to evaluate the protein content. Cell lysate was subjected to 12% SDS-polyacrylamide and gel electrophoresis was performed. Samples were transferred to nitrocellulose membranes (GE Healthcare Europe GmbH, Freiburg, Germany). The membranes were then blocked for 1 hour at room temperature with 5% skim milk in Tris-Buffered Saline with Tween-20 (TBST). Finally, the membranes were washed three times with TBST before being probed with specific primary antibodies against different apoptotic molecules including poly(ADP-ribose) polymerase (PARP) with 1 : 1000 dilution in TBST and β-actin overnight at 4 °C. All protein expression levels were calculated relative to β-actin expression. The membranes were washed three times before being exposed to a suitable secondary antibody in TBST with 1 : 1000 dilution for 1 hour at room temperature. After three further washes with TBST, an ECL substrate enhancer (SuperSignal West Femto Substrate, Thermo Scientific, US) was applied to the membrane to generate a protein band and any detectable changes were captured using the molecular ChemiDoc XRS system (Bio-Rad, US). ImageJ software was then used to analyse the band density. As an internal control for protein expression, the level of β-actin was assessed.

### Negative staining for transmission electron microscopy of the phage particles

A formvar carbon-coated 200 mesh grid was glow discharged at 10 mA for 2 minutes. The phage was applied on the surface of the grid and incubated for 1 minute. The excess phage was blotted on absorbent paper. Next, the grid was washed with filtered deionized water and then blotted on absorbent paper. The grid was floated on a drop of filtered deionized water for 3 minutes and blotted on absorbent paper. Next, the grid was floated on a drop of 1% glutaraldehyde for 5 minutes and blotted on absorbent paper. The phage was negatively stained using 1% uranyl acetate to the grid for 4 minutes and blotted on absorbent paper. Finally, the images were finally obtained using a transmission electron microscope (JEOL JEM-2010, Japan).

### Bioinformatics analysis

Affymetrix HG-U133Plus2.0 DNA microarray (Platform GPL570) of 30 HCC samples (GSE6222, GSE40367, GSE45435 and GSE45436) and 13 normal liver (GSE7307, GSE14951 and GSE88839) composition were retrieved from Gene Expression Omnibus (GEO). All data were obtained from tissues without any therapy. The gene expression analysis was obtained by a comparison of HCC samples to the healthy liver samples. The robust multi-array average (RMA) algorithm through a custom brain array chip description file (CDF, ENTREZG, V19) was used to calculate the quantile normalization background adjustment and summarized as previously described.^22^ For the investigation of differential gene expression, *P* values were calculated with Linear Models for Microarray (limma) data in *R*.^23^

### Statistical analysis

All data were represented as mean ± SD. For statistical analysis, we used independent *t*-test and one-way ANOVA test. Statistical significance was expressed as *p* values of <0.05, 0.01 and 0.001. All statistical analyses were invented using GraphPad prism 9.5.0 software.

## Results

### HCC expresses receptors for the RGD4C ligand and TRAIL death receptors

Two crucial components are required when using tumour-targeted TPA carrying the TRAIL gene. First, targeting cells must express α_v_β_3_ or α_v_β_5_ integrins (RGD4C binding receptors) on the cell surface. These receptors selectively bind to the targeted TPA particles, allowing for their internalisation. Second, the targeting cell must express death receptors. The TRAIL selectively promotes apoptosis by interacting with death receptors TRAIL-R1 (or DR4) and TRAIL-R2 (or DR5), which are excessively expressed in different types of cancer cells.^24^ In addition, low or no expression of decoy receptors should be observed on the targeted cell. The TRAIL is known to bind to three decoy receptors: TRAIL-R3 (or DcR1), TRAIL-R4 (or DcR2), and OPG.^24^ Only the TRAIL binding to DR4 or DR5 can trigger apoptotic signaling.^25^

We assessed the expression of integrin receptors in the human Huh-7 and HepG2 HCC cell lines. Both cell lines were stained with integrin heterodimer-specific antibodies and subjected to flow cytometric analysis. Fig. 1A shows that the percentage of Huh-7 cells that expressed the α_v_β_3_ and α_v_β_5_ integrins was 2.61% and 55.1%, respectively, The HepG2 cell expressed the α_v_β_3_ and α_v_β_5_ integrins at 4.81% and 59.1%, respectively. Therefore, Huh-7 and HepG2 cells can serve as human HCC models for tumor-targeted TPA. Next, we investigated the five members of the TRAIL receptors by flow cytometric analysis. Fig. 1B shows that only the death receptor DR5 was expressed in both Huh-7 (66.3%) and HepG2 (95.4%). In addition, the decoy receptors, DcR1 and DcR2, were not expressed on both HCC cell surfaces, but OPG was expressed in both Huh-7 (35.9%) and HepG2 (56.8%). We considered that the high expression of α_v_β_5_ and DR5 in both HCC cell lines is adequate for tumour-targeted delivery of therapeutic genes and selective cancer cell death by the TPA particles. We further analysed the expression of α_v_β_3_ and α_v_β_5_ integrins and five members of TRAIL binding receptors in normal LX-2 cells used as a model for normal liver cells.^26,27^ The results showed low or no expression of α_v_β_3_ or α_v_β_5_ integrins and death receptors in LX-2 cells (ESI Fig. 2†). These results suggest that normal hepatic cells are not harmed by the tumour-targeted TPA delivering the *TRAIL* gene.

### Expression of the TRAIL by TPA-*tmTRAIL* plasmid triggered apoptosis in HCC cells

We designed and generated a TPA plasmid carrying a full length human *tmTRAIL* gene under a *CMV* protomer transgene expression cassette flanked by the *ITR cis*-elements from AAV-2. These elements facilitate the efficient and sustained expression of transgenes.^28^ The efficacy of the TPA-*tmTRAIL* DNA construct in mediating the TRAIL gene expression and induction of apoptosis was tested in Huh-7 and HepG2 HCC cells.

**Fig. 2:**
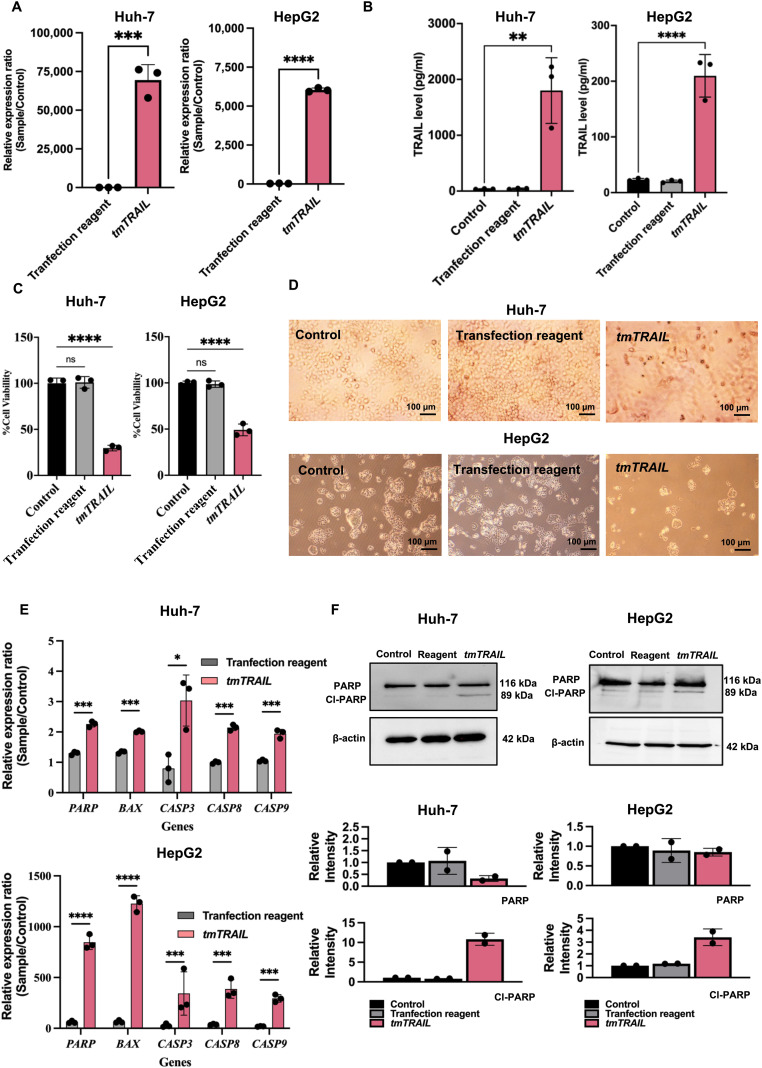
TPA-*tmTRAIL* construct-mediated TRAIL expression and cell death in HCC. Human HCC Huh-7 and HepG2 cells at 60% confluency were transfected with the TPA-*tmTRAIL* plasmid. The untreated cells and transfection reagent alone were used as controls. Cells were transfected with the TPA plasmid for 48 hours at 37 °C. TRAIL expression from the transfected cells was determined by qPCR (A) and ELISA (B). Cell viability was measured using the MTT assay (C) and cell morphology was imaged under a light microscope (D). Images were taken using 10× objective lens. Evaluation of apoptotic gene expression by qPCR (E). Translational expression of PARP was measured by western blotting to confirm the result at the transcriptional level (F). Data were normalised to control untreated cells and shown as fold change relative to control. All results are shown as mean ± SD. All experiments were conducted in triplicate, except for western blot analysis, which was performed in duplicate. *P < 0.05, **P < 0.01 and ***P < 0.001.

At 48-hour post-transfection, cell lysates and media were harvested to determine the TRAIL expression by RT-qPCR for the mRNA expression and ELISA for the detection of the TRAIL protein. The high expression of the TRAIL was observed in TPA-*tmTRAIL*-transfected HCC cells, both in Huh-7 and HepG2. TRAIL RNA expression levels in Huh-7 and HepG2 were 69431- and 6307-fold higher compared to control cells, respectively, (Fig. 2A) with no TRAIL RNA levels in Huh-7 and HepG2 transfected with the TPA plasmid without *tmTRAIL* (ESI Fig. 6A†). Then, by ELISA, 1801 and 209.7 pg mL^−1^ of the TRAIL protein were detected from the cell lysates of Huh-7 and HepG2, respectively (Fig. 2B). Next, we determined the bioactivity of the recombinant TRAIL by measuring cell viability with the MTT assay at 48-hour post-transfection. As shown in Fig. 2C–D and ESI Fig. 6B and C,† the cell viability of both *tmTRAIL*-transfected HCC cells was significantly reduced to 29.7% (Huh-7) and 49.1% (HepG2) of untreated cells (control). Furthermore, we examined the expression of the apoptotic genes in the transfected cells. The results show that the transcriptional levels of apoptotic genes including *BAX*, *CASP3*, *CASP8*, *CASP9* and *PARP* were significantly higher in the TPA-*tmTRAIL* plasmid transfected cells (Fig. 2E), compared to the cells treated with the transfection reagent only as a control group. We further confirmed the expression at the protein level with cleaved PARP by western blot analysis. As shown in Fig. 2F and ESI Fig. 3,† both HCC cell lines in the TPA-*tmTRAIL* transfected group showed a significantly higher level of cleaved PARP (Cl-PARP).

### TPA vector efficiently targets and delivers transgene expression to HCC cells

**Fig. 3:**
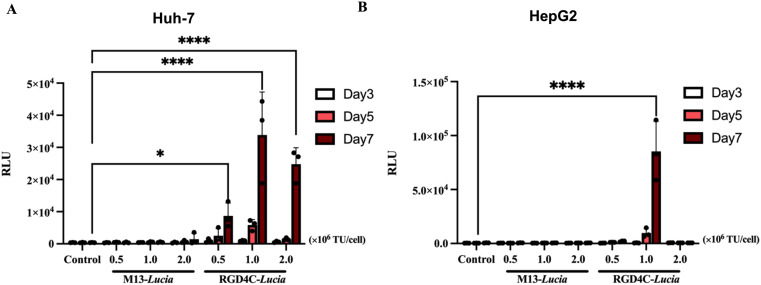
Efficient targeted gene delivery to human HCC cells following treatment with the RGD4C.TPA-*lucia* particle. Using three different transducing units (TUs) per cell of the vectors (0.5, 1.0 and 2.0 × 10^6^ TU per cell), human Huh-7 (A) and HepG2 (B) cells were treated with either the tumor-targeted RGD4C.TPA-*lucia* or the non-targeted M13.TPA-*lucia* particles. Additionally, untreated cells served as control. Secreted luciferase (*lucia*) activity was assessed in the culture medium of cells on days 3, 5, and 7 post transduction. Results from representative experiment's triplicate wells are shown as mean ± SD. *P < 0.05, **P < 0.01, ***P < 0.001 and ****P < 0.0001.

The TPA platform targets tumour cells by exhibiting the double cyclic RGD4C ligand on its pIII minor coat proteins of (RGD4C.TPA).^10^ In this study, we showed that α_v_β_5_ integrins, the RGD4C binding partner, are highly expressed on both HepG2 and Huh-7 HCC cell surfaces. We then evaluated the targeted gene delivering efficacy of the RGD4C.TPA particle to HCC cells. We used the TPA particle carrying the secreted luciferase reporter gene, TPA-*lucia*, to test RGD4C.TPA-mediated gene delivery efficacy. Different transduction units of tumour-targeted TPA-*lucia* (RGD4C.TPA-*lucia*) or non-targeted TPA-*lucia* (M13.TPA-*lucia*) with 0.5, 1.0 and 2.0 × 10^6^ TU per cell were used to transduce Huh-7 and HepG2 cells. After transduction, on days 3, 5, and 7, we monitored *lucia* activity from the cultured medium. The results demonstrated a considerable increase of the *lucia* activity in both HCC cell lines treated with RGD4C.TPA-*lucia* in a dose- and time-dependent manner (Fig. 3A and B). Remarkably, the M13.TPA-*lucia* treated group from both HCC cell lines did not show any increase of the *lucia* activity. These findings suggest that our RGD4C.TPA facilitates effective and selective gene delivery to human HCC cells.

### Expression of the TRAIL by tumour-targeted TPA-*tmTRAIL* particle-triggered apoptosis in HCC cells

**Fig. 4:**
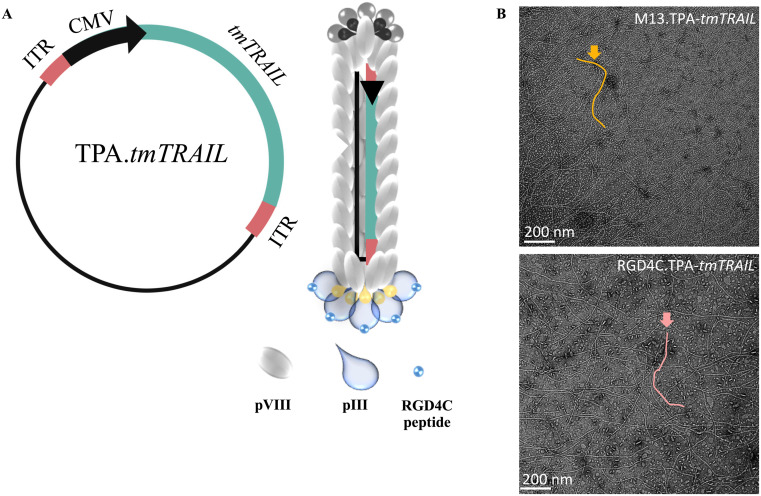
Generation of the RGD4C.TPA-*tmTRAIL* particle. The *tmTRAIL* encoding RGD4C targeting TPA construct and virions are shown schematically (A). To serve as a targeting ligand that binds specifically to the α_v_β_3_ or α_v_β_5_ integrin receptors, the TPA exhibits five copies of the double cyclic RGD4C ligand on its pIII minor coat proteins. The TPA genome was genetically modified by insertion of a transgene cassette flanked by AAV-2 *ITR cis*-elements. (B) Electron microscopy images of targeted RGD4C.TPA-*tmTRAIL* and control non-targeted TPA-*tmTRAIL* particles are shown.

After confirming that the RGD4C.TPA particle efficiently delivered the *lucia* gene to both HCC cell lines, we replaced the *lucia* gene with the transmembrane form of the human TRAIL gene (*tmTRAIL*) and generated the TPA particle carrying the *tmTRAIL* gene (RGD4C.TPA-*tmTRAIL*) as shown in Fig. 4A. The non-targeted particle, M13.TPA-*tmTRAIL* lacking the tumor targeting RGD4C ligand, was used as control for targeting.

**Fig. 5:**
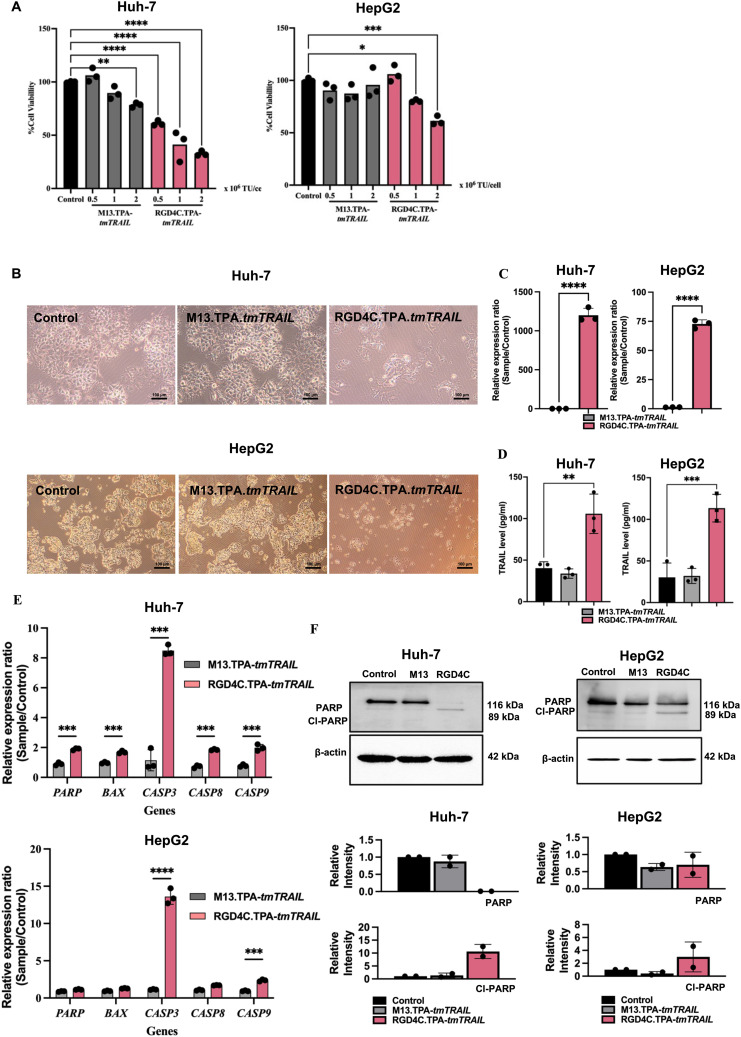
HCC cell death mediated by TPA-*tmTRAIL* particles. (A) Human HCC Huh-7 and HepG2 cells at 60% confluency were transduced with RGD4C.TPA-*tmTRAIL* particles with three different transducing units (TUs) per cell of the vectors (0.5, 1.0 and 2.0 × 10^6^ TU per cell). The untreated cells and non-targeted M13.TPA-*tmTRAIL* were used as controls. Cells were treated with the TPA particles for 16–18 hours at 37 °C. Measurement of TRAIL mRNA expression by RT-qPCR (A) and protein levels by ELISA (B) in lysate of HCC cell lines. Data were normalised to untreated cells and shown as fold change relative to control. Cell viability was measured using the MTT assay on day 7 post-transduction (C). Huh-7 and HepG2 cells were observed using a microscope (D). Images were taken using 10× objective lens. Expression of apoptotic death-related molecules by RT-qPCR (E) and translational expression of cleaved PARP was measured by western blotting to confirm the result from transcriptional level (F). Data were normalised to control (untreated cell) and shown as fold change relative to control. All results are shown as mean ± SD. All experiments were conducted in triplicate, except for western blot analysis, which was performed in duplicate. *P < 0.05, **P < 0.01, ***P < 0.001 and ****P < 0.0001.

Following production, the structure and size of the TPA particles were validated and visualised by transmission electronic microscopy (TEM) (Fig. 4B). Both targeted and non-targeted TPA particles are approximately 1000 nm long and similar in diameter. We treated HCC cell lines with increasing doses of TPA-*tmTRAIL* particles, ranging 0.5, 1.0 and 2.0 × 10^6^ TU per cell for five days, and cell viability was determined using the MTT assay. Notably, treatment with RGD4C.TPA-*tmTRAIL* significantly induced cell death in a dose dependent manner in both HCC cells; however, the most significant cell death was observed in HepG2 cells at doses of either 1.0 × 10^6^ or 2 × 10^6^ TU per cell (Fig. 5A). Treatment with M13.TPA-*tmTRAIL* did not cause any cell death in all treatment regimens. Analysis of cell morphology at 1.0 × 10^6^ TU per cell (Fig. 5B) showed that cells from the control (untreated) and non-targeted M13.TPA-*tmTRAIL* treated groups maintained normal morphology while the cells from RGD4C.TPA-*tmTRAIL* treated group were eradicated. Therefore, we chose the biotherapeutic dose of RGD4C.TPA-*tmTRAIL* at 1.0 × 10^6^ TU per cell for further studies. Thus, we determined the TRAIL expression from the cell lysates of TPA-*tmTRAIL* transduced HCC cells using by both RT-qPCR and ELISA. The results demonstrated a significant increase in the TRAIL expression in both Huh-7 and HepG2 cells treated with RGD4C.TPA-*tmTRAIL* (Fig. 5C and D). We further measured apoptotic cell death markers in transduced HCC cells using RT-qPCR. There was a significant increase in *BAX, CASP3*, *CASP8*, *CASP9* and *PARP* expression in Huh-7 cells treated with the RGD4C.TPA-*tmTRAIL* (Fig. 5E). In the HepG2 cell, we found that *CASP3* and *CASP9* mRNA levels were significantly increased in cells treated with RGD4C.TPA-*tmTRAIL* compared to the non-targeted vector (Fig. 5E). We further confirmed the expression at the protein level with cleaved PARP through western blot analysis. As shown in Fig. 5F and ESI Fig. 5,† both HCC cell lines treated with RGD4C.TPA-*tmTRAIL* particles showed a significantly higher level of cleaved PARP (Cl-PARP). These findings collectively demonstrate that RGD4C.TPA-*tmTRAIL* is a promising therapeutic particle for use in the treatment of human HCC.

### Tumour-targeted gene delivery efficacy of TPA in HCC tumourspheres and its translational relevance

**Fig. 6:**
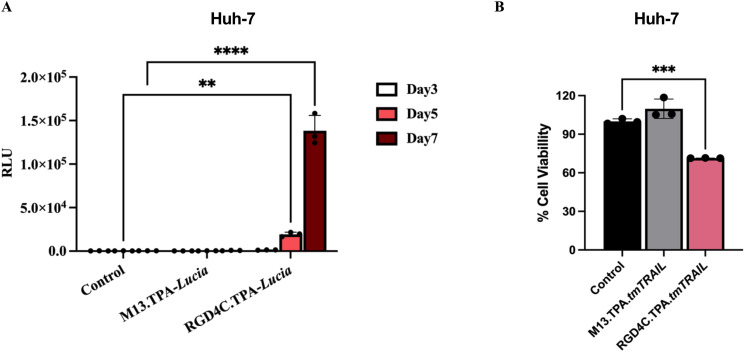
TPA particle-mediated targeted gene delivery in Huh-7 spheroid culture. (A) Secreted luciferase (*lucia*) activity from cultured media of transduced Huh-7 spheroid culture. TPA-*lucia* at 1 × 10^6^ TU per cell was used for transduction. (B) Cell viability of transduced Huh-7 spheroid culture. TPA-*tmTRAIL* at 1 × 10^6^ TU per cell was used for transduction. Cell viability was determined on day 7 post transduction using the MTT assay. All results are shown as mean ± SD. All experiments were conducted in triplicate. *P < 0.05, **P < 0.01 and ***P < 0.001.

**Fig. 7:**
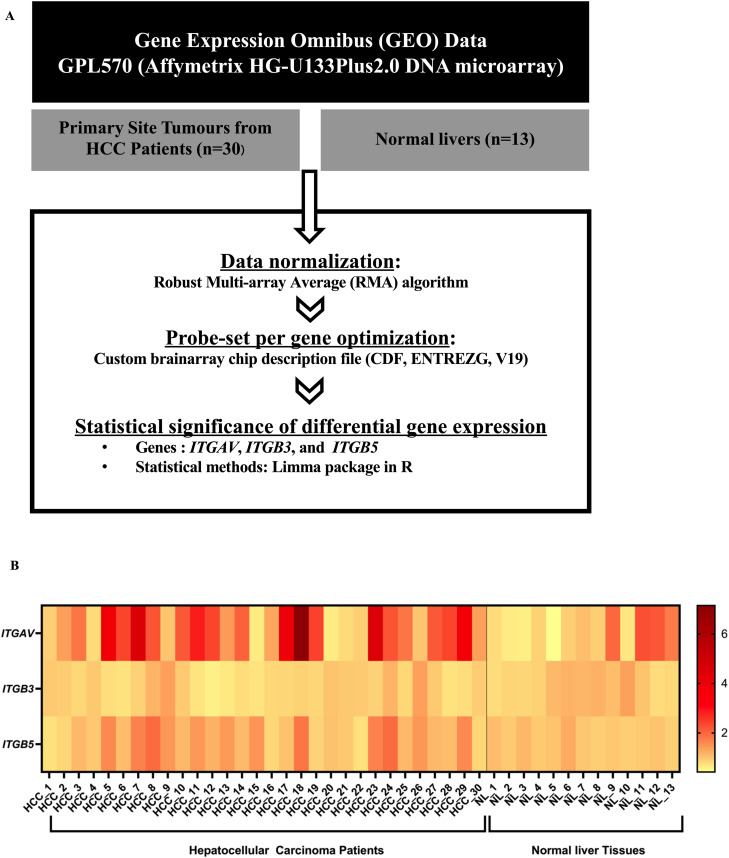
Expression of integrin receptors on primary human HCC tissues. (A) Diagram of comparative expression analysis. (B) Heatmap of α_v_, β_3_ and β_5_ receptors identified in primary human HCC tissues at the primary site (*n* = 30). The HCC samples were compared with normal liver tissues (*n* = 13).

The tumoursphere, or spheroid, model is vital in cancer research as it enables the cultivation of cancer cells in a manner that closely resembles their natural state within tumours. This three-dimensional structure encourages the preservation of cellular heterogeneity, which is a crucial driver of tumour growth and resistance to therapy.^29^ Spheroids allow the cells to interact with each other in a more physiologically relevant manner compared to two-dimensional cultures, ultimately contributing to the development of more effective treatments.^30–33^ In this experiment, we established a spheroid model based on the Huh-7 cells cultured on a polyHEMA (non-adherent polymer) coated plate for 48 hours to allow the cells to grow as spheroids. We first examined the targeted gene delivery efficacy of the TPA particle carrying the *lucia* gene. After spheroid formation, both targeted RGD4C.TPA-*lucia* and non-targeted TPA-*lucia* were added to the Huh-7 spheroid culture plate and *lucia* activity was monitored over a seven-day period from cultured media. The results demonstrated a considerable increase in the *lucia* activity only from the spheroid culture treated with RGD4C.TPA-*lucia*, in a time-dependent manner (Fig. 6A). We then transduced the Huh-7 spheroid with TPA-*tmTRAIL* particles and cell viability was assessed on day 7 post transduction. Fig. 6B shows that treatment of RGD4C.TPA-*tmTRAIL* in Huh-7 spheroids significantly induced cell death at all dosages tested. No cell death was observed in the non-targeted M13.TPA-*tmTRAIL*. Altogether, we showed that the tumour-targeted RGD4C.TPA particles effectively deliver genes to a three-dimensional tumour model. To further support the RGD4C.TPA application against HCC in a clinical context, the HCC must express α_v_β_3_ or α_v_β_5_ integrins. Therefore, we employed meta-analysis to determine these receptors in patient samples. We compared the α_v_, β_3_ and β_5_ integrin receptor expression between HCC and normal liver tissues using differential gene expression from the Gene Expression Omnibus (GEO) database, following the diagram as shown in Fig. 7A. The heatmap demonstrated that the expression of α_v_ and β_5_ integrin receptors, but not β_3_, were significantly upregulated in almost HCC tissues as analysed by comparative expression analysis (Fig. 7B and ESI Table 2†). These data are consistent with the results of integrin expression in Huh-7 and HepG2 cells (Fig. 1A). In conclusion, the results demonstrated that the RGD4C.TPA-*tmTRAIL* particle is a promising tool for HCC treatment.

## Discussion

In this study, we evaluated the efficacy of the tumour-targeted TPA (RGD4C.TPA) particle in the targeted delivery of therapeutic genes to HCC. Our data indicate that the particle effectively and selectively delivered transgenes to HCC cells. The delivery of the TRAIL gene to HCC cells by the RGD4C.TPA-*tmTRAIL* particle resulted in TRAIL expression and subsequently induced HCC cell death.

Bacteriophage-based vectors offer several advantages over other viral and non-viral vectors. Since phages have been co-evolved with bacteria over millions of years, leading to a lower likelihood of eliciting strong immunogenicity and have little human tropism or pathogenicity.^10^ Engineering of the bacteriophage gene is straightforward, allowing for the customization of its genetic material. Furthermore, filamentous phage-based vectors also provide very high payload capacity comparing to other viral vectors, for example, AAVs, as payload capacity is a major challenge. In our previous study, we showed that the phage-based vector can accommodate large transgenes while maintaining the ability to deliver the gene to cancer cells.^16,34^ Furthermore, modifications of bacteriophage capsids, through genetic engineering or chemical conjugation, are easily manipulated with a minimal impact on the overall structure and stability of the particle.^35^ In terms of production, phage-based vectors are superior to other popular eukaryotic viral vectors. These vectors can be produced within 24 hours and purification is straightforward using PEG/NaCl precipitation. The production process does not require sophisticated equipment for production and purification and is cost-effective as it does not necessitate expensive production materials.^10,12^ In term of safety, bacteriophages have been used to treat bacterial infection in humans. Several studies have demonstrated success in utilising phage therapy for antibiotic-resistant bacterial infections.^36,37^ Our previous pre-clinical investigations showed that our phage-based vector treatment is non-harmful to organs and the entire experimental subjects.^10,16^ Corresponding to this study, we used the LX-2 cell line, representing normal liver cells to validate the safety and specificity of the vector. LX-2 cell remained unharmed when treated with RGD4C.TPA-*tmTRAIL* (Fig. 5A and ESI Fig. 5†).

As bacteriophages does not normally infect mammalian cells, we genetically modified the phage capsid to exhibit a specific ligand targeting a specific receptor present on the target cells or tissue.^15^ The pIII minor coat proteins of the M13 phage were observed with a double-cyclic RGD4C peptide ligand. The ligand specifically binds to α_V_β_3_ and α_V_β_5_ integrin receptors on the tumour cells. When the TPA particle reaches the target cancer cells, the RGD4C ligands observed on the TPA pIII capsid will bind to the heterodimer α_v_β_3_ or α_v_β_5_ integrins, and cell surface receptors were overexpressed in most tumour types including HCC, but barely expressed in normal cells. This interaction initiates the internalisation of TPA particles into cancer cells and their engulfment inside endosomes. Some of the TPA particles can escape from the endosome, while the others end up being degraded in lysosomes. The escaping particles release the transgene cassette from their capsids, and the transgene is translocated into the nucleus. The transgene cassette, flanked by ITR elements from AAV2, forms concatemers in the nucleus, proceeding with transgene expression.^10,38^ The TPA capsid can be further modified by decorating the pVIII capsid with the endosomal escape peptide (H5WYG), which can increase the transgene expression.^39^ Therefore, HCC cells must exhibit these receptors on their surface. In this study, we showed that HCC cell lines express α_V_β_5_ on their surface, and we demonstrated that our TPA vector effectively targets and delivers the TRAIL transgene to both the cell lines. There was no quantifiable expression of transgenes, both *lucia* and TRAIL, observed in the non-targeted (M13) vector group, indicating the specificity of the RGD4C ligands. To translate this vector into clinical applications, primary tumour cells of a patient must also express both integrins on their surface. Weiler *et al.* reported that overexpression of the α_V_ integrin in HCC patients was associated with the poor clinical outcome by promoting HCC cell invasion.^40^ Another study demonstrated that elevated levels of α_V_ in HCC also facilitate HCC cell migration.^41,42^ Moreover, earlier reports indicated that the protein expression of the β_5_ integrin is higher in HCC tissues compared to adjacent tissues. Additionally, overexpression of the β5 integrin promotes HCC cell migration and carcinogenesis both *in vitro* and *in vivo.*^43^ Furthermore, upregulation of α_V_β_5_ induced HCC proliferation and drove HCC angiogenesis.^20^ From our meta-analysis result, we demonstrated that the gene expression of *ITGAV* and *ITGB5*, analysed from obtained samples in GEO, was significantly upregulated in HCC tissues compared with normal liver tissue. To underscore the safety of using the particle, we previously demonstrated that a panel of normal human primary cells from various histological origins do not express or have extremely low expression of the RGD4C binding partners, α_V_β_3_ and α_v_β_5_ integrin receptors.^20^ These findings suggest that TPA is an appropriate delivery method for therapeutic genes to HCC.

The application of TRAIL protein-based therapies has been hindered by challenges such as its short half-life and off-target effects on non-cancerous tissues. Off-target effects may occur if the TRAIL protein binds to receptors on cells other than the intended cancer cells. While the TRAIL preferentially induce apoptosis on cancer cells, it may interact with death receptors (DR4 and DR5) on normal cells, leading to apoptosis in healthy tissues. There have been no reports on severe systemic toxicity of the TRAIL protein in murine, non-human primates when using it as a therapeutic agent,^44,45^ but study has shown that the TRAIL protein can induce apoptosis in normal human hepatocytes *in vitro*.^46^ Another limitation has been related to its poor pharmacokinetic profile, as it has a very short half-life in mammals; this is likely due to its small molecular weight and the instability of its non-covalently linked trimeric structure, both of which result in its rapid elimination.^47^ In clinical trials with the TRAIL protein, the half-life of the TRAIL is approximately only 0.5–1 h post intravenous injections.^5,48^

The recombinant human TRAIL has shown promising results in the treatment of hepatocellular carcinoma in preclinical studies.^1^ Nevertheless, the TRAIL has a relatively short half-life and its rapid clearance from the bloodstream can limit its effectiveness in reaching HCC tumours.^3–5^ In clinical trials, the use of the recombinant TRAIL in HCC treatment has been primarily focused on the evaluating its safety and efficacy when administered either alone or in combination with other therapies. Some of the limitations observed in these trials included low overall response rates to the recombinant TRAIL in HCC and systemic side effects associated with its administration in HCC patients. TRAIL-based gene therapy involves introducing genes encoding the TRAIL or its receptors directly into cancer cells. This prompts the cancer cells to produce the TRAIL, subsequently triggering apoptosis in themselves due to the TRAIL. This approach can help overcome the short half-life of the TRAIL.^3–5^ By utilising gene therapy to deliver the TRAIL gene, cancer cells can continuously produce the TRAIL protein, overcoming the short half-life of the TRAIL and achieving a more optimal concentration of the TRAIL at the tumour site. This sustained production of the TRAIL enables prolonged and more effective therapeutic effects.^7–9,25^ TRAIL signaling is mediated by interaction with two cell surface death receptors, DR4 and DR5. The expression of DR4 and DR5 in cancer cells can vary widely depending on the type of cancer and the specific characteristics of tumour. DR4 and DR5 can also be expressed in cells within the tumour microenvironment, such as stromal cells and endothelial cells.^49^ In contrast, DR4 and DR5 are typically expressed at low levels in normal cells.^1,2^ Our findings showed that Huh-7 and HepG2 overexpress DR5 receptors. Moreover, the expression of other decoy receptors, DcR1 and DcR2, was not detected in HCC. This can make the HCC cells more sensitive to TRAIL apoptosis-inducing therapies. We demonstrated that the overexpression of transmembrane TRAIL-mediated RGD4C.TPA promoted apoptosis in both HCC cell lines. Despite the presence of osteoprotegerin (OPG), the soluble form of the TRAIL decoy receptor member, as observed in this study, both HCC cell models remain sensitive to TRAIL-induced apoptosis. A previous study described that OPG has a much weaker binding affinity to DR5 (≤400 nM) compared to the TRAIL (≤2 nM) at physiological temperatures.^50^ Therefore, OPG cannot compete with the TRAIL to bind to the DR5 receptor in this instance.

Alternative 3D culture models have been developed in recent years. In cancer research, 3D models are recognised as an intermediate model between 2D cultures and *in vivo* experiments, capable of more accurately representing certain characteristics of *in vivo* tumours, such as cell–cell and ECM–cell interactions, a necrotic center, gradients of oxygen, nutrients, and pH decreasing from periphery.^51–53^ Our results demonstrated the effective treatment of RGD4C.TPA-*tmTRAIL* particles to the HCC spheroids, an *in vivo* mimic model, resulting in HCC spheroid cell death. This highlights another advantage of the bacteriophage-based vector over other viral vectors. Many viral vectors have a relatively large particle, for example, lentivirus (80–100 nm) and AAV (20–25 nM), limiting their accessibility into tumour masses. In contrast, filamentous phage-based vectors (TPA) have very small diameters (5 nm). Furthermore, our TPA vector can be administered through non-invasive systemic therapeutic routes. Both teams and our research partners have demonstrated its effectiveness in a variety of pre-clinical human cancer models, including melanoma, breast, prostate, brain, and pancreatic cancer, and chondrosarcoma.^15,54–59^ Next, we plan to further investigate the efficacy and safety of this tumour-targeted TPA-*tmTRAIL* particles in the animal model of hepatocarcinoma.

## Conclusions

In conclusion, our study establishes targeted gene therapy as a new HCC treatment strategy to address the limitations of present treatments. Superior TPA particles were shown to be a promising selective and efficient method for hepatocellular carcinoma gene therapy. An important point is that eukaryotic cells cannot be infected by native bacteriophages. As a result, this platform may represent an additional HCC treatment strategy with the ability to provide specificity and efficacy of the therapeutic gene approach.

## Author contributions

Conceptualization: AH, PP, PS, and KS; methodology: AH, PP, PS, KS, and AC; formal analysis: PP, PS, and KS; investigation: PP, PS, KS, BW and AC and; resources: AH, PP, KS, and PK; data curation: AH, PP, and KS; writing—original draft preparation: PS; writing—review and editing: AH, KS and PS; visualization: AH, KS and PS. All authors have read and agreed to the published version of the manuscript.

## Conflicts of interest

AH and KS are the inventors of two patent applications related to this work. These patents have been licensed to GENSAIC, USA. The remaining authors declare that they have no conflict of interest.

## Supplementary Material

NR-016-D3NR05660K-s001
