# Supplementary material for: Bacteriophage-based particles carrying the TNF-related apoptosis-inducing ligand (TRAIL) gene for targeted delivery in hepatocellular carcinoma
Source: Nanoscale. 2024 Feb 23;16(13):6603–17. doi: 10.1039/d3nr05660k (PMC10977282; doi:10.1039/d3nr05660k)
Supplement: NR-016-D3NR05660K-s001 [file NR-016-D3NR05660K-s001.pdf]

Electronic Supplementary Material (ESI) for Nanoscale.
This journal is © The Royal Society of Chemistry 2024

Supplementary Table 1. Primers

**Group of genes**	**Name**	**Sequence (5'-3'): Forward (F); Reverse (R)**
Housekeeping gene	*Human ACTB*	F: GTAAAGACCTCTATGCCAACA<br>R: GGACTCATCGTACTCCTGCT
Therapeutic gene	*TNFSF10* (Human TRAIL)	F: GAAGACCTCAGAAAGTGGC<br>R: GACCAGCTCTCCATTCCTA
Apoptotic relative genes	*Caspase 3*	F: TGGTTCATCCAGTCGCTTTG<br>R: CATTCTGTTGCCACCTTTCG
	Caspase 7	F: CCAATAAAGGATTTGACAGCC<br>R: GCATCTGTGTCATTGATGGG
	Caspase 8	F: GCCTCCCTCAAGTTCCT<br>R: CCTGGAGTCTCTGGAATAACA
	Caspase 9	F: CGAACTAACAGGCAAGCAGC<br>R: ACCTCACCAAATCCTCCAGAAC
	BID	F: GCTTCCAGTGTAGACGGAGC<br>R: GTGCAGATTCATGTGTGGATG
	BAX	F: CCTGTGCACCAAGGTGCCGGAACT<br>R: CCACCCTGGTCTTGGATCCAGCCC
	Bcl-2	F: CATGCTGGGGCCGTACAG<br>R: GAA CCGGCACCTGCACAC
	XIAP	F: AACCTTGTGATCGTGCCT<br>R: ACCCTGGATACCATTTAGC
	PARP	F: AAGGCGAATGCCAGCGTTAC<br>R: GCACTCTTGGAGACCATGTCA

Supplementary Table 2. Comparative expression analysis

Gene	Encoding protein	Adjusted P.value	logFC	Fold change (HCC:normal liver)
ITGAV	Integrin αv	0.022379	0.930177791	1.905510808
ITGB3	Integrin β3	0.263627	-0.123141995	0.918185787
ITGB5	Integrin β5	0.027514	0.320521683	1.24878203

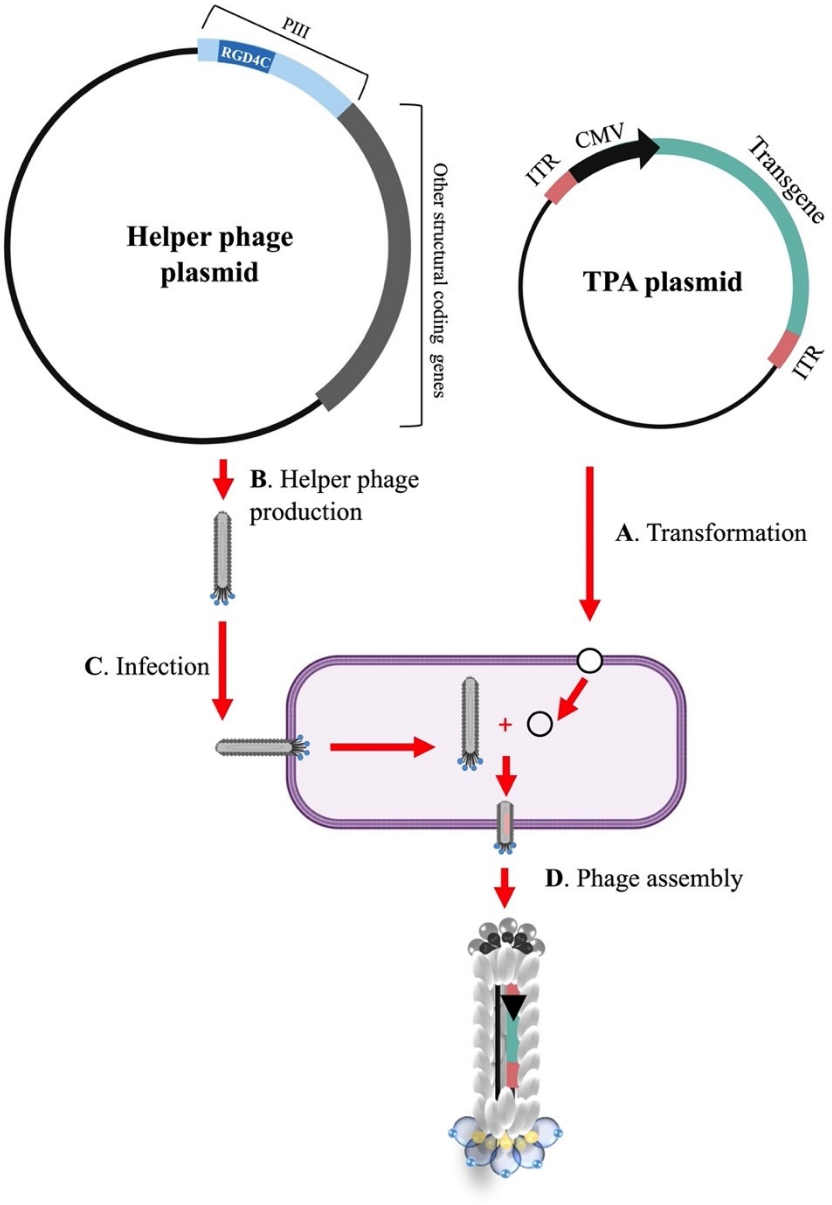

Supplementary Figure 1 TPA particle production involves the incorporation of two essential components: the TPA plasmid and the helper phage plasmid. The TPA plasmid harbours a mammalian transgene cassette flanked by AAV-2 ITRs, while the M13 helper phage plasmid carries the tumour-targeted RGD4C peptide insertion mutation on the pIII minor coat protein gene. The genome of M13 helper phage also encompasses other structural genes necessary for phage assembly. To generate the TPA particle, the TPA plasmid is transformed into F' competent *E. coli* hosts (A) which are subsequently infected with RGD4C.M13 helper phage. This leads to the encapsidation of the AAV DNA cassette using bacteriophage capsid proteins (B-C). The resulting particle exhibits a tumour-targeted bacteriophage and exclusively contains the AAV DNA transgenic cassette expressing a gene of interest (D).

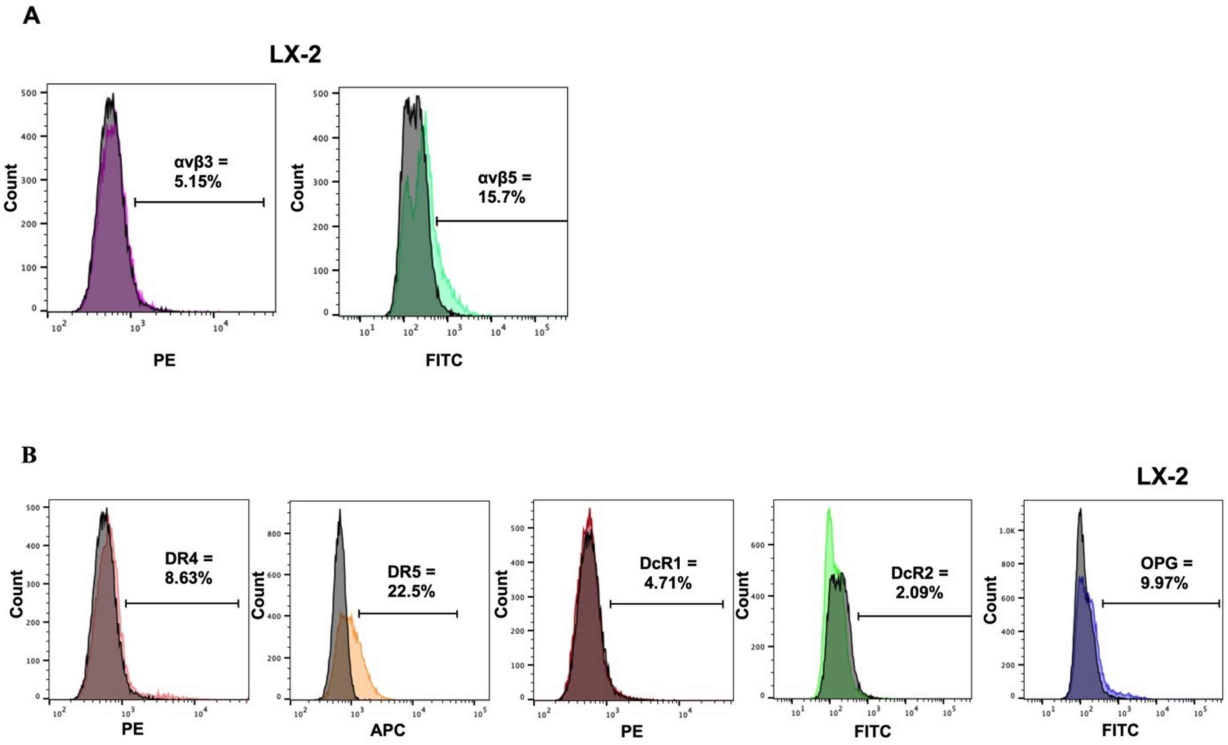

Supplementary Figure 2 Expression of integrins ($\alpha_v\beta_3$, and $\alpha_v\beta_5$) and TRAIL death receptors on human liver cell line (LX-2). Histograms of flow cytometric analysis of integrins (A) and TRAIL receptors (B). The black histograms served as the unstained or isotype control staining cells.

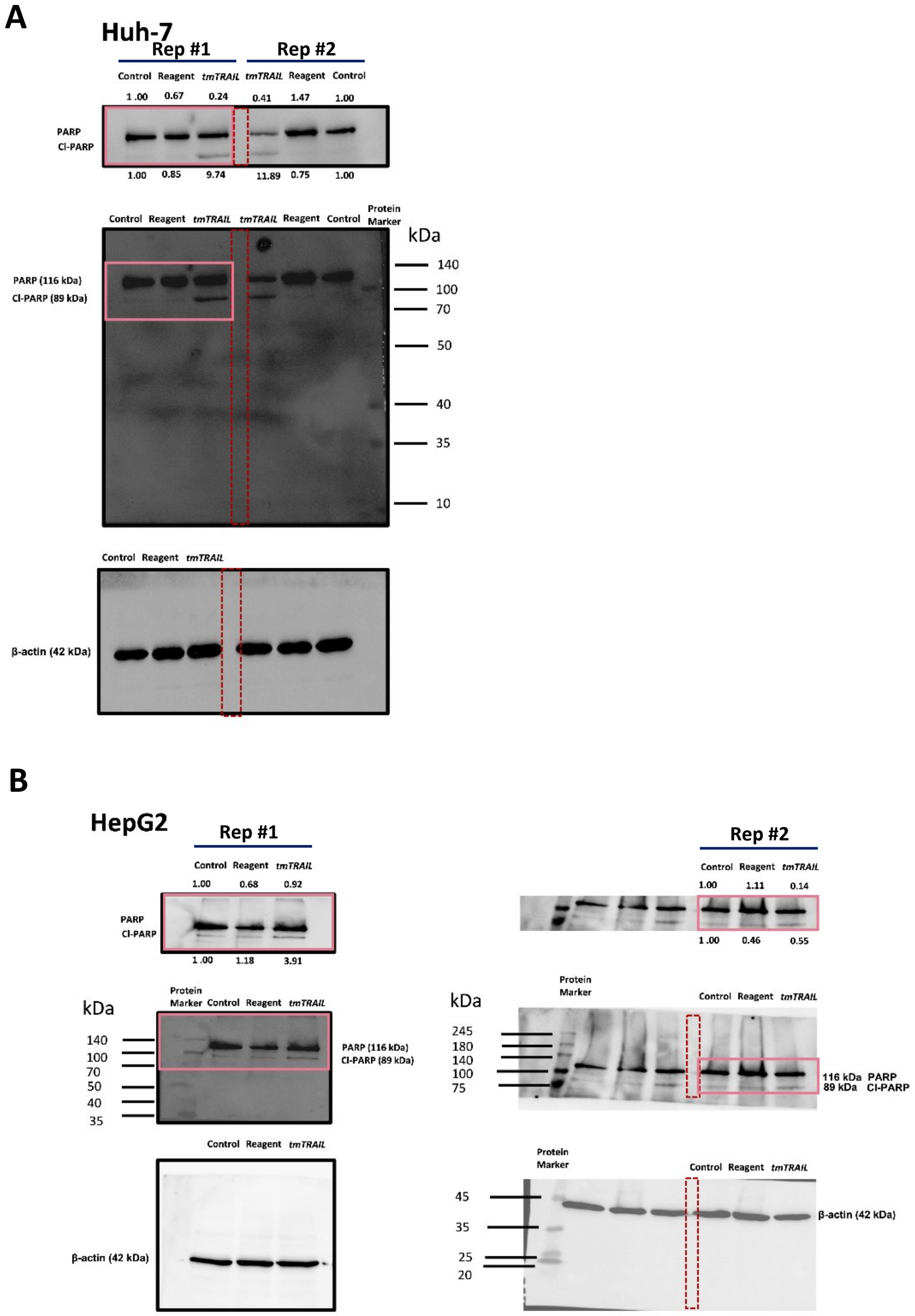

Supplementary Figure 3. The raw data from the representative western blot analysis (whole blot) in Figure 2F depicts the results obtained from Huh-7 **(A)** and HepG2 **(B)** cells transfected with TPA-*tmTRAIL* plasmid. The top panel represents cropped data, the middle panel shows the entire membrane alongside molecular weight protein markers and the bottom panel exhibits the same membrane re-probed with anti-β-actin. The data enclosed within the pink rectangular area represents the data shown in Figure2F, while the results outside this boundary correspond to the second replicating experiment. The discontinuous red rectangular demarcates results the first (Rep#1) and second (Rep#2) replications. Untreated cells (control) and transfection reagent (reagent) were used as control groups. Cell lysates were subjected to 12% gel SDS-PAGE and western blotting. The membrane was probed with anti-PARP antibody. The antibody detects both PARP and cleaved PARP (Cl-PARP). The membrane was stripped and re-probed with anti-β-actin (internal control) antibody. Band densities were quantified using ImageJ. The expression levels of PARP and Cl-PARP were normalised to β-actin and represented as fold changes in comparison to control group. The fold change values were indicated adjacent to the band (PARP; above the band, Cl-PARP; below the band). Thermo Scientific Spectra Multicolor Broad range protein marker was used in this experiment.

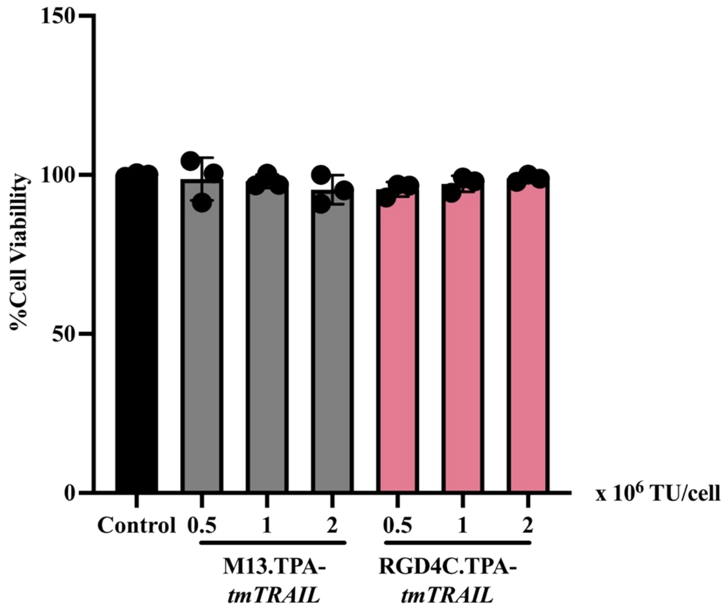

Supplementary Figure 4. Normal liver cells were not adversely affected by RGD.TPA-*tmTRAIL* particles. Human LX-2 cells at 60% confluency were transduced with RGD.TPA-*tmTRAIL* using three different transducing unites (TU) per cell of the vectors (0.5 ,1.0 and 2.0 × 10^6 TU/ cell). Untreated cells and non-targeted TPA-*tmTRAIL* were used as control groups. The cells were treated with the TPA particles for 16-18 hours at 37°C. Cell viability was measured by using MTT assay at 7 days post-transduction. All results are shown as mean ± SD. Experiment was conducted in triplicate.

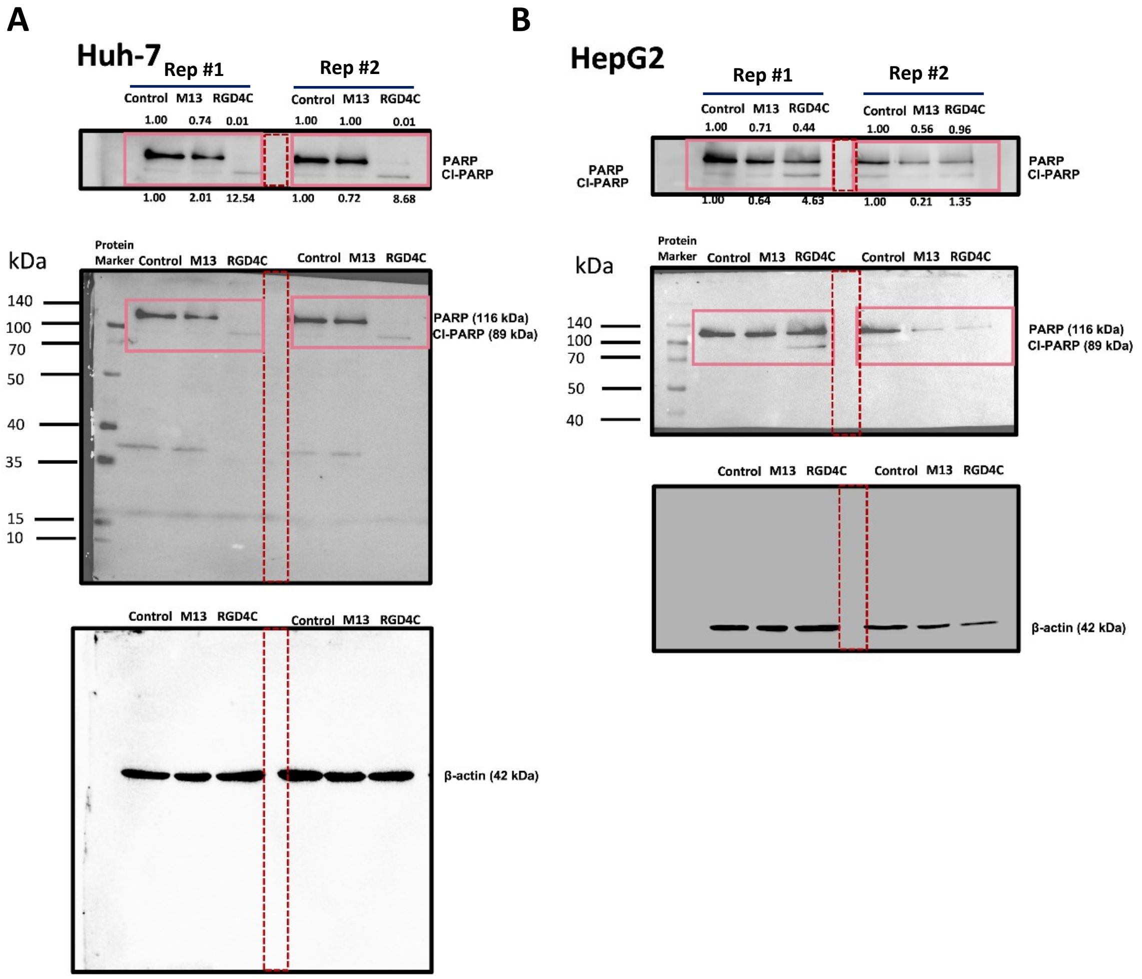

Supplementary Figure 5. The raw data from the representative western blot analysis (whole blot) in Figure 5F depicts the results obtained from Huh-7 **(A)** and HepG2 **(B)** cells transduced with tumour-targeted RGD4C.TPA-*tmTRAIL* (RGD4C) particle. Untreated cells (control) and non-target M13.TPA-*tmTRAIL* (M13) treatment were used as control groups. TPA particles at a concentration of $1x10^6$ TU per cell were used for cell transduction. The top panel represents cropped data, the middle panel shows the entire membrane alongside molecular weight protein markers and the bottom panel exhibits the same membrane re-probed with anti-β-actin. The data enclosed within the pink rectangular area represents the bands of PARP and Cl-PARP. The discontinuous red rectangular demarcates results the first (Rep#1) and second (Rep#2) replications. Cell lysates were subjected to 12% gel SDS-PAGE and western blotting. The membrane was probed with anti-PARP antibody. The antibody detects both PARP and cleaved PARP (Cl-PARP). The membrane was stripped and re-probed with anti-β-actin (internal control) antibody. The band densities were assessed using ImageJ. The expression of PARP and Cl-PARP were normalised to β-actin and represented as fold changes compared to control group. The fold change values were indicated adjacent to the band (PARP; above the band, Cl-PARP; below the band). Thermo Scientific Spectra Multicolor Broad range protein marker was used in this experiment.

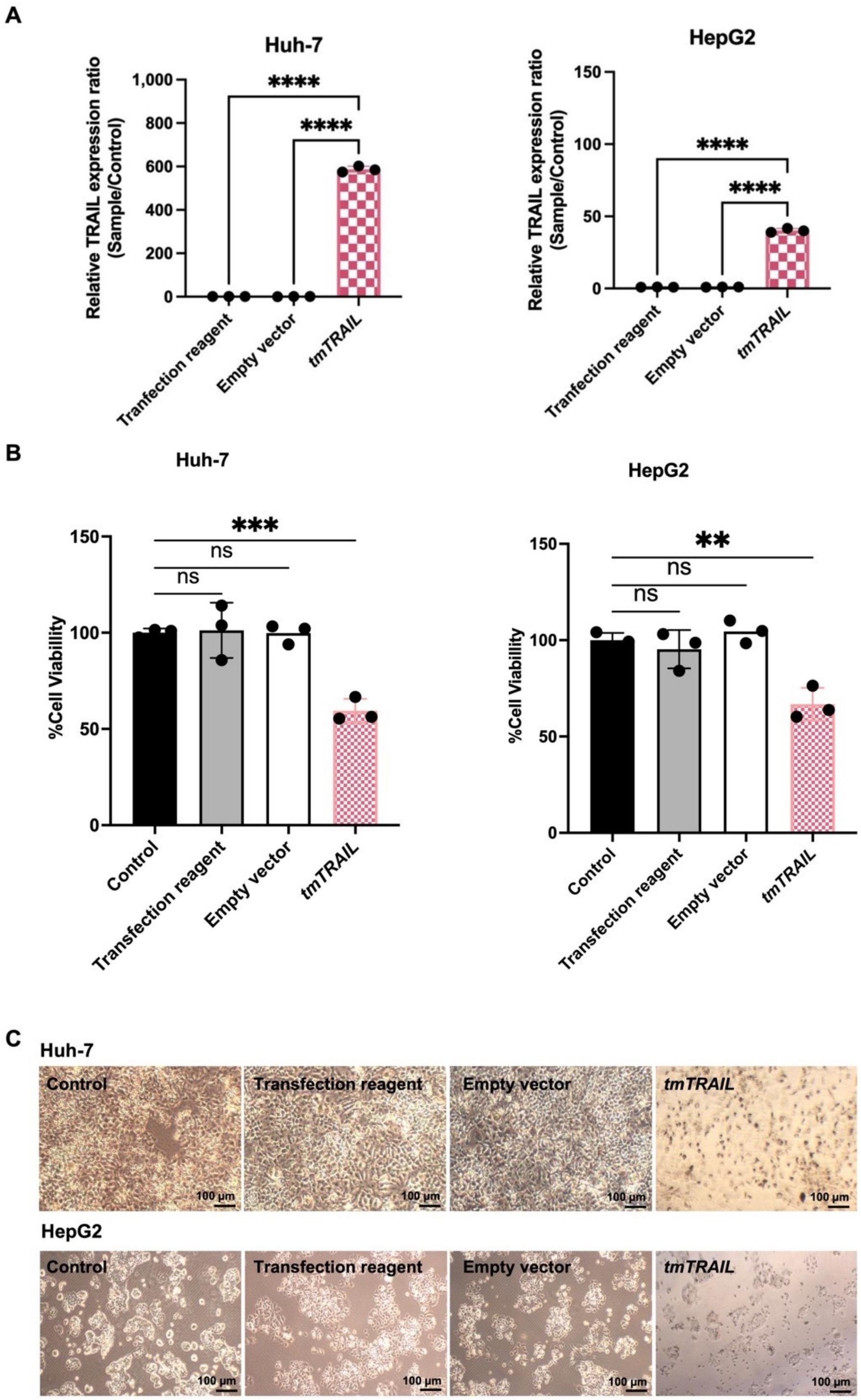

Supplementary Figure 6 Transfection of TPA plasmid without tmTRAIL gene (empty vector) show no toxicity to HCC. Human HCC Huh-7 and HepG2 cells at 60% confluency were transfected with TPA-*tmTRAIL* plasmid. The untreated cells, transfection reagent alone and empty vector were used as controls. Cells were transfected with TPA plasmid for 48 hours at 37°C. TRAIL expression from the transfected cells was determined by RT-qPCR (A). Cell viability was measured using the MTT assay (B), and cell morphology was imaged under a light microscope (C). Data were normalised to control untreated cells and shown as fold change relative to control. All results are presented as mean ± SD. All experiments were conducted in triplicate, $^{**}P < 0.01$, $^{***}P < 0.001$ and $^{****}P < 0.0001$.
